# Prognostic role of translocator protein and oxidative stress markers in chronic lymphocytic leukemia patients treated with bendamustine plus rituximab

**DOI:** 10.3892/ol.2014.2817

**Published:** 2014-12-19

**Authors:** ANIELLO DE ROSA, SILVIA ZAPPAVIGNA, MARIA ROSARIA VILLA, SALVATORE IMPROTA, ELENA CESARIO, LUCIA MASTRULLO, MICHELE CARAGLIA, PAOLA STIUSO

**Affiliations:** 1 Local Sanitary Agency, Naples 3 Southern, Torre del Grecco, Naples I-80059, Italy; 2 Department of Biochemistry, Biophysics and General Pathology, Second University of Naples, Naples I-80138, Italy; 3 Hematology Unit, San Gennaro Hospital, Naples I-80131, Italy

**Keywords:** apoptosis, chronic lymphocytic leukemia, prognostic factor, translocator protein, NO, TBARS

## Abstract

Principally located in the outer mitochondrial membrane, the translocator protein (TSPO) is an 18-kDa transmembrane protein that is a key component of the mitochondrial permeability transition pore. TSPO is associated with a number of biological processes, including apoptosis, the regulation of cellular proliferation, porphyrin transport and heme biosynthesis, immunomodulation, anion transport and the regulation of steroidogenesis. Thus, numerous studies have proposed TSPO as a promising target for novel therapeutic agents, particularly for the treatment of cancer. In the present study, the response of 30 consecutive chronic lymphocytic leukemia (CLL) patients to bendamustine and rituximab treatment was evaluated according to TSPO expression levels. Furthermore, thiobarbituric acid reactive substances (TBARS) and nitric oxide (NO) levels, as well as caspase-3 activity were determined. Compared with the lymphocytes of healthy donors, the 30 consecutive CLL patients exhibited increased TSPO expression levels, decreased TBARS and NO levels and reduced caspase-3 activity. Six months after the treatment commenced, the TSPO/mitochondria ratio resembled that of the healthy controls in 24/30 CLL patients. In addition, an increase in TBARS and NO levels, two markers of oxidative stress, and a potentiation of caspase-3 activity in all responder patients was observed. Notably, the six patients who appeared to be resistant to treatment also displayed higher TSPO levels, and lower caspase-3 activity and TBARS levels. These data indicate that TSPO expression may be a molecular prognostic factor in CLL patients.

## Introduction

Previously termed the peripheral benzodiazepine receptor, the translocator protein (TSPO) is a key element of the mitochondrial permeability transition pore (MPTP), which is a multiprotein complex located at the contact sites between the inner and outer mitochondrial membranes of various different cell types, including cells of the hematopoietic system. TSPO is physically associated with the backbone of MPTP via its adenosine nucleotide translocase and voltage-dependent anion channel (VDAC) (1). Consistent with its localization in the MPTP, TSPO has been implicated in various mithochondrial functions, such as the control of respiration, the modulation of inner membrane ion channel activities, as well as the regulation of apoptosis, cell proliferation and a number of other associated processes (2). It has previously been demonstrated that overexpression of TSPO inhibits apoptosis induced by free radicals or ultraviolet light. Concordantly, TSPO ligands have been demonstrated to induce caspase-3 (3) and -9 activation, the cytosolic release of cytochrome *c* and cause an increase in the production of reactive oxygen species (ROS), resulting in the activation of p38 mitogen activated protein kinase and the opening of the MPTP (4). Furthermore, Carayon *et al* (5) demonstrated that TSPO expression correlated with H_2_O_2_ cytotoxicity resistance in hematopoietic cell lines (5); following the transfection of Jurkat cells with human TSPO complementary DNA, H_2_O_2_ resistance increased compared with wild-type cells. These results indicate that TSPO may aid in preventing free radical-induced cellular damage of mitochondria and may regulate cell apoptosis in the hematopoietic system.

Previously, TSPO and its ligands have been highlighted as potential targets for the development of novel anticancer agents (6). TSPO is highly expressed in various cancer cell types, including colon (7), brain (8), breast (9), ovary (10–11) and liver (12) cancer, and its expression is particularly high in organs involved in steroidogenesis (13). Although additional studies are required to improve the understanding of the biological functions of TSPO, clear evidence exists regarding its involvement in steroid biosynthesis. Furthermore, TSPO is key in cancer cell growth (14–17), with Maaser *et al* (18) demonstrating that specific TSPO ligands are able to induce apoptosis and cell cycle arrest in colorectal cancer cells.

Additionally, TSPO is regarded as a potential prognostic factor in cancer. In particular, it has been reported that lymphoma cell lines (19–20) and myeloid/lymphoid cells obtained from leukemia patients (5,20) express high levels of TSPO, and ovarian, hepatic and colonic carcinomas, as well as glioma (7–8,10–12) have demonstrated increased TSPO densities compared with the corresponding healthy tissue. In specific cases, TSPO expression correlates with the grade of the tumor malignancy and patient survival (21); for example, relatively high levels of TSPO density were observed in more rapidly proliferating breast cancer cells (22) and more aggressive breast cancer phenotypes (23).

CLL is characterized by the accumulation of mature malignant cluster of differentiation (CD) 5^+^ B-lymphocytes. Despite the use of numerous agents in the treatment of CLL, patients may develop mechanisms of resistance (24). In order to stratify patients according to their potential to respond to targeted therapy, personalized targeted therapy has been proposed; however, this requires the identification of novel (bio)markers.

In the present study, the role of TSPO as prognostic factor in CLL patients was investigated by evaluating the response to bendamustine and rituximab treatment according to TSPO expression. In addition, thiobarbituric acid reactive substance (TBARS) and nitric oxide (NO) levels, as well as caspase-3 activity, were analyzed in the lymphocytes of healthy donors compared with CLL patients.

## Materials and methods

### Preclinical characteristics, treatment strategy and peripheral blood mononuclear cell (PBMC) isolation

The present study included 10 healthy blood bank donors and 30 patients from the Hematology Unit of San Gennaro Hospital (Naples, Italy). The patients enrolled in the present study were diagnosed with CLL according to the following criteria: Monotypic expansion of lymphoid cells (≥15×10^3^/μl), morphologically consistent with CLL (small lymphocytes), in the blood for ≥60 days prior to treatment; >30% lymphocytes in the bone marrow; and normal renal (creatinine, <2.0 mg/dl) and hepatic (bilirubin, <2.0 mg/dl) function. No patients had previously been treated for CLL and no patients had previously received four cycles of bendamustine plus rituximab. The treatment strategy administered in the present study was intravenous rituximab (375 mg/m^2^) every 28 days, followed by intravenous bendamustine (90 mg/m^2^) on the next day, for two consecutive days. This treatment regimen was continued until the occurrence of disease progression or unacceptable levels of toxicity.

The response criteria used was that previously defined by the National Cancer Institute Working Group (25). Complete remission (CR) was defined as the absence of all palpable disease and the return of the blood counts to within the following normal ranges: Neutrophils, >1.5×10^3^/ml; platelets, >100×10^3^/ml; hemoglobin, >11 g/dl; and bone marrow aspirate lymphocyte percentage*,* <30%. Partial remission (PR) was defined as a 50% decrease in palpable disease accompanied by a 50% improvement in all abnormal blood parameters. Patients were considered to be responsive to the treatment if a PR of ≥6 months was observed and resistant to the treatment if a PR of <6 months or no remission (NR) were observed. PBMCs were isolated from blood samples obtained prior to (T0), one week after (T1) and six months after (T6) the commencement of treatment. The PBMCs were isolated using Ficoll density gradient centrifugation, as previously described (26) and stored at -80°C for use in the following experiments. In addition, sera was collected from the patients at the abovementioned time periods and stored at −20°C for use in the following experiments. This study was approved by the ethics committee of San Gennaro Hospital and written informed consent was obtained from all patients.

### Analysis of TSPO protein expression levels by flow cytometry

The PBMCs were fixed for 20 min in a 3% (w/v) paraformaldehyde (PFA) solution and permeabilized for 10 min with 0.1% (w/v) Triton X-100 in phosphate-buffered saline (PBS) at room temperature. To prevent non-specific interactions occurring between antibodies, the cells were treated with 5% bovine albumin serum (BSA) in PBS for 2 h and incubated with a specific mouse monoclonal antibody raised against TSPO [cat. no. SAB1405525; dilution, 1:1,000 in blocking solution; 3% (w/w) BSA in 0.1% Tris-buffered saline-Tween; Sigma-Aldrich, St. Louis, MO, USA] for 2 h at 37°C. Following multiple washes, the cells were incubated with a secondary IgG goat anti-mouse monoclonal antibody (cat. no. A-11001; Alexa Fluor^®^ 488; Life Technologies, Grand Island, NY, USA) diluted 1:1,000 in blocking solution for 1 h at room temperature. The samples were subsequently washed twice and, for each sample, 10,000 cells were counted using a FACSCalibur™ flow cytometer (BD Biosciences, Franklin Lakes, NJ, USA) and CellQuest™ software (BD Biosciences). As TSPOs are expressed on mitochondria, the cellular mitochondrial content of TSPO was determined as follows: The cells were fixed with 3% PFA, labeled with 10^−7^ mol/l nonyl acridine orange for 15 min at room temperature, washed twice in PBS and analyzed using flow cytometry. Thus, the mitochondrial TSPO density was determined by calculating the number of TSPO sites per unit of mitochondrial mass.

### Nitrite assay

Nitric oxide (NO) is a molecular mediator of numerous physiological processes, including vasodilation, inflammation, thrombosis, immunity and neurotransmission. Thus, a number of methods exist, which analyze NO levels in biological systems. Under physiological conditions, NO is rapidly converted into the stable end products nitrite (NO2-) and nitrate and subsequently the hematic concentrations are frequently assessed as an index of systemic NO production. Therefore, nitrite levels were measured using the Griess reaction, as previously described by Gomez-Monterrey et al (27).

### TBARS levels

The PBMC samples were incubated with 0.5 ml of 20% acetic acid (pH 3.5) and 0.5 ml of 0.78% aqueous thiobarbituric acid solution. The mixture was heated to 95°C for 45 min and centrifuged at 1,600 × g for 5 min. To quantify the amount of TBARS in the supernatant fractions, spectrophotometry was performed at an absorbance of 532 nm (27) and data were expressed as TBARS/serum protein in μM/μg. Data are presented as the average of triplicate measurements from duplicate experiments.

### Caspase-3 activity

The BD ApoAlert™ Caspase-3 Fluorogenic assay kit (BD Biosciences Clontech, Palo Alto, CA, USA) and a fluorescent microplate reader (Applied Biosystems Life Technologies, Foster City, CA, USA) were used to determine the caspase-3 activity levels, as previously described (28).

### Statistical analysis

Statistical analyses were conducted by performing an analysis of variance with Neumann-Keul’s multiple comparison test or the Kolmogorov-Smirnov test, as appropriate. Analyses of the differences between the CR and NR patients were performed using the Mann-Whitney U test for non-parametric independent and continuous variables and all data are expressed as the mean ± standard deviation. ^*^P<0.01 indicates a statistically significant difference between the control and CLL patients.

## Results

### Patient characteristics and clinical response to therapy

A total of 30 patients were enrolled from the Hematology Unit of San Gennaro Hospital. The pretreatment clinical characteristics of the 30 patients are indicated in Table I. Among the 30 enrolled patients, 67% were male and the median age was 73 years, with 48% patients aged ~65 years and 52% patients aged ≥75 years. Compared with the healthy patients, the CLL patients exhibited a significantly higher median white blood cell count (75,000/mm^3^; P=0.004), a marginally lower median hemoglobin level (11.5 g/dl; P=0.05) and a marginally higher median platelet count (180,000/mm^3^; P=0.05).

The CLL patients received four cycles of treatment with bendamustine plus rituximab. Patients were considered to be responsive to the treatment if a PR of ≥6 months was observed and resistant to the treatment if a PR of <6 months or NR were observed. Following six months of treatment with bendamustine plus rituximab, 12/30 (40%) patients achieved CR, eight (26%) patients achieved PR, six (20%) patients achieved no remission (NR) and four (13%) patients succumbed to the disease (Table II).

### Modulation of TSPO expression

To evaluate the use of TSPO as a therapeutic target and prognostic marker, the response of CLL patients to bendamustine and rituximab treatment was determined according to TSPO expression. As TSPO is located on the mitochondria, the cellular mitochondrial content was determined by performing flow cytometry analysis; furthermore, TSPO density at the mitochondrial level was calculated by the number of TSPO sites per unit of mitochondrial mass.

**Figure 1:**
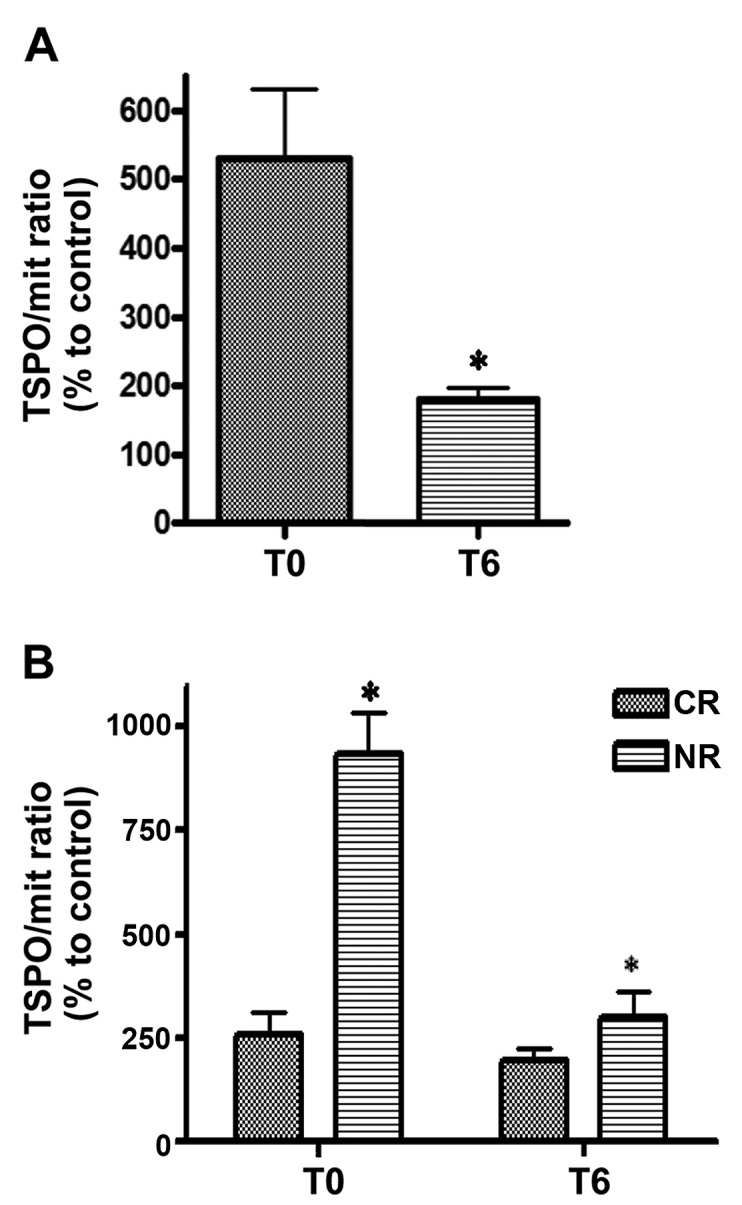
Evaluation of the variation in translocator protein (TSPO) expression in 30 patients with CLL. TSPO density at the mitochondrial level was obtained by calculating the number of TSPO sites per unit of mitochondrial mass. (A) Changes in TSPO expression in 30 CLL patients with respect to healthy patients at T0 and T6. (B) Changes in TSPO expression in CR and NR patients with respect to healthy patients at T0 and T6. Error bars represent the standard deviation of the mean. ^*^P<0.01, NR at T0 vs. CR at T0, NR at T0 vs. CR at T6, NR at T0 vs. NR at T6 and NR at T6 vs. CR at T6. CR, complete remission; NR, no remission; Mit, mitochondria; PBR, peripheral-type benzodiazepine receptor; T0, before treatment commenced; T6, six months after treatment commenced.

TSPO expression was evaluated at T0 and T6. Compared with the lymphocytes of the healthy participants, the leukemic cells of the 30 CLL patients exhibited an increased level of TSPO, normalized for mitochondrial expression (Fig. 1A).

Six months after the treatment commenced, a decrease in the TSPO/mitochondria ratio occurred, resembling that of the healthy controls in 24/30 CLL patients (Fig. 1A). CR patients exhibited low TSPO levels at T0, which were marginally reduced following six months of treatment compared with the NR group (Fig. 1B). Notably, the six patients who were resistant to treatment (NR patients) displayed significantly (P<0.01) higher TSPO levels compared with CR patients at T0; furthermore, the NR TSPO levels were significantly (P<0.01) reduced following six months of therapy (Fig. 1B). Thus, the results indicate an inverse trend in the TSPO levels between the two groups of patients.

### Modulation of serum levels of NO, TBARS and caspase-3 activity

The present study evaluated TBARS and NO levels, two markers of oxidative stress, in the lymphocytes of 30 CLL patients at T0, T1 and T6.

**Figure 2:**
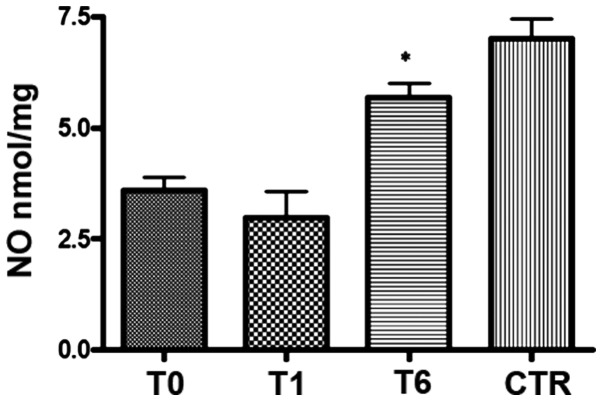
Serum NO protein expression level variation in 24 responder patients compared with healthy patients using an enzyme spectrophotometric assay. Error bars represent the standard deviation of the mean. ^*^P<0.01 vs. T0, T1 and CTR. CTR, healthy patients; NO, nitric oxide; T0, before treatment; T1, one week after treatment commenced; T6, six months after treatment commenced.

**Figure 3:**
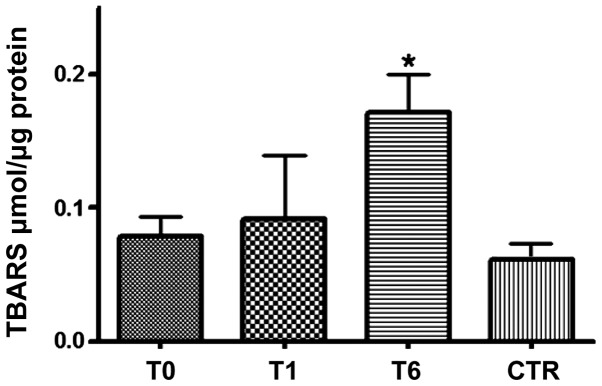
TBARS protein expression level variation in 24 responder patients compared with healthy patients using an enzyme spectrophotometric assay. Error bars represent the standard deviation of the mean. ^*^P<0.01 vs. T0, T1 and CTR. CTR, healthy patients; T0, before treatment; T1, one week after treatment commenced; T6, six months after treatment commenced; TBARS, thiobarbituric acid reactive substance.

**Figure 4:**
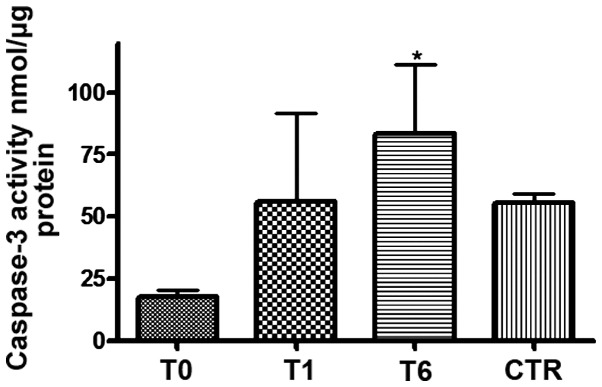
Caspase-3 activity variation in 24 responder patients compared with healthy patients using an enzyme spectrophotometric assay. Error bars represent standard deviation of the mean. ^*^P<0.01 vs. T0 and CTR. CTR, healthy patients; T0, before treatment; T1, one week after treatment commenced; T6, six months after treatment commenced.

At T0, lower levels of NO (Fig. 2) and marginally higher levels of TBARS (Fig. 3) were identified in the CLL patients compared with the healthy controls. Bendamustine plus rituximab therapy at the T1 timepoint did not significantly change the serum NO levels (Fig. 2) but did slightly increase the TBARS level (Fig. 3); however, NO (Fig. 2) and TBARS (Fig. 3) mean serum levels were significantly (P<0.0001) increased in all responder patients (24/30) at T6. TBARS but not NO levels exceeded the mean values recorded in healthy subjects. In addition, apoptosis was evaluated by detecting caspase-3 activity in the CLL patients. Prior to treatment (T0), low caspase-3 activity was identified in the CLL patients compared with the healthy donors; however, one week after treatment commenced, an increase in caspase-3 activity was observed in all of the responder patients (24/30) and an additional increase was observed six months after the commencement of treatment (Fig. 4).

Of note, the six patients who appeared to be resistant to treatment displayed lower caspase-3 activity and TBARS levels six months after therapy (data not shown). These data indicate again that the increase in NO, TBARS and caspase-3 activity levels recorded following six months of treatment may be predictive of a response to therapy.

## Discussion

TSPO is a component of the MPTP (with other protein constituents, such as VDAC) and is involved in a number of biological processes, including apoptosis, the regulation of cellular proliferation, porphyrin transport and heme biosynthesis, immunomodulation, anion transport, and the regulation of steroidogenesis. In addition, TSPO facilitates the regulation of the release of apoptotic factors into the cytosol; this correlation between TSPO, and the occurrence of programmed cell death and apoptotic onset was identified by studying the role of TSPO in the MPTP (29). TSPO causes prolonged opening of the MPTP and the release of apoptotic factors, such as Smac, cytochrome *c* and apoptosis*-*inducing factor (30,31), from the mitochondria into the cytosol, resulting in osmotic swelling of the mitochondrial matrix, and dysregulation of ATP synthesis and oxidative phosphorylation. Ultimately, apoptosis and necrotic signaling cascades are activated and cell death occurs (32), highlighting the correlation between TSPO and the protection of cells from apoptosis. Notably, previous studies have identified an association between TSPO and cancer; for example, it was demonstrated that TSPO overexpression induced by transfection protects lymphocytes against ultraviolet light-induced cell apoptosis (33), and TSPO expression was correlated with the ability of breast cancer cells to grow in severe combined immunodeficient mice (34). Furthermore, it was demonstrated that TSPO positively correlates with proliferation rate but inversely correlates with spontaneous apoptosis rates in various glioma cell lines. In patients exhibiting tumors with TSPO overexpression, TSPO may contribute to a poor prognosis by mediating proliferative and/or apoptosis-protective effects. However, these functions of TSPO may be reversed by the administration of TSPO-specific exogenous ligands in a variety of tumor types (14–18); furthermore, increased binding capacities of TSPO-specific ligands have been reported in colorectal cancer (18) and a variety of different tumor types (14–16). However, TSPO protein overexpression was only directly determined in astrocytoma and breast cancer. In addition, a study of 86 astrocytoma patients identified that TSPO protein expression correlated with the tumor grade (9) and, in a small number of breast cancer samples and cell lines, TSPO protein expression correlated with malignant cancer (21).

In the present study, TSPO expression levels were evaluated in 30 CLL patients treated with bendamustine plus rituximab. Following six months of treatment, 12/30 (40%) patients achieved CR, eight (26%) patients achieved PR, six (20%) achieved no remission (NR) and four (13%) succumbed to the disease. TSPO levels were higher in leukemic cells compared with the lymphocytes of healthy individuals at T0; however, six months after treatment commenced, the present study identified a decrease in the TSPO/mitochondria ratio in 24/30 CLL patients to resemble that of the healthy controls. In addition, CR patients exhibited low TSPO levels at T0 compared with NR patients. Thus, the results of the present study indicate an inverse trend in TSPO expression levels in the two groups of patients.

The role of NO varies in cancer biology and is dependent on a number of factors; NO may be involved in the promotion or prevention of tumor occurrence, depending on the time of exposure to NO, the tumor microenvironment and the concentration of NO. Low concentrations of NO (range, 1–30 nM) produced high levels of cyclic guanosine monophosphate, promoting tumor angiogenesis and the proliferation of endothelial cells, and a wider range of NO concentrations (range, 0–100 nM) corresponded to an increase in the activity of the proliferative and anti-apoptotic Akt and Erk-dependent pathways in tumor cells (35,36), appearing to enhance angiogenesis and protect tumor cells from apoptosis. Thus, at NO concentrations of 0–100 nM, the molecules activated by NO are considered to be correlated with poor prognosis events in cancer. By contrast, higher NO levels (>300 nM) appeared to promote apoptosis and anti-tumor activity.

In the present study, a spectrophotometric assay was performed to determine the serum NO levels of CLL patients treated with bendamustine plus rituximab. Six months after (T6) treatment commenced, responsive patients exhibited a strong increase in NO and TBARS levels, as well as a potentiation of caspase-3 activity. Notably, the six patients who were resistant to treatment exhibited lower caspase-3 activity and TBARS levels. These data again indicate that the increase in NO and TBARS levels, as well as caspase-3 activity levels, recorded at T6 may be predictive of a response to therapy. Notably, in responsive patients, the increased NO levels possibly had an anti-tumor and pro-apoptotic role, as demonstrated by the corresponding increase in caspase-3 activity.

In conclusion, these data indicate that TSPO expression may be a molecular prognostic marker in CLL patients. In addition, TSPO may represent a useful therapeutic target for increasing treatment efficacy in CLL patients.

## Figures and Tables

**Table I:** Pretreatment clinical characteristics (n=30).

Variable	Value
Median age, years	73
Age distribution, %	
65 years	48
75–80 years	50
≥80 years	2
Gender distribution, %	
Male	67
Female	33
Median white blood cell count,/mm^3^	78,000
Median hemoglobin level, g/dl	11.5
Median platelet count,/mm^3^	180,000

**Table II:** Clinical response to therapy (n=30).

Clinical response	Patients, n (%)
Complete remission	12 (40)
Partial remission	8 (26)
No remission	6 (20)
Mortality	4 (13)

## Abbreviations

CLL: chronic lymphocytic leukemia
CR: complete remission
MPTP: mitochondrial permeability transition pore
NO: nitric oxide
NR: no remission
PR: partial remission
PBMC: peripheral blood mononuclear cells
TBARS: thiobarbituric acid reactive substances
TSPO: translocator protein
VDAC: voltage-dependent anion channel
